# The Insula: A Brain Stimulation Target for the Treatment of Addiction

**DOI:** 10.3389/fphar.2019.00720

**Published:** 2019-07-02

**Authors:** Christine Ibrahim, Dafna S. Rubin-Kahana, Abhiram Pushparaj, Martin Musiol, Daniel M. Blumberger, Zafiris J. Daskalakis, Abraham Zangen, Bernard Le Foll

**Affiliations:** ^1^Translational Addiction Research Laboratory, Centre for Addiction and Mental Health, Toronto, ON, Canada; ^2^Department of Pharmacology, University of Toronto, Toronto, ON, Canada; ^3^Department of Psychiatry, Faculty of Medicine, University of Toronto, Toronto, ON, Canada; ^4^Qunuba Sciences, Toronto, ON, Canada; ^5^Ironstone Product Development, Toronto, ON, Canada; ^6^Temerty Centre for Therapeutic Brain Intervention, Campbell Family Mental Health Research Institute, Centre for Addiction and Mental Health, Toronto, ON, Canada; ^7^Department of Life Sciences and the Zlotowski Center for Neuroscience, Ben-Gurion University of the Negev, Beer-Sheva, Israel; ^8^Addictions Division, Campbell Family Mental Health Research Institute, Centre for Addiction and Mental Health, Toronto, ON, Canada; ^9^Institute of Medical Sciences, University of Toronto, Toronto, ON, Canada; ^10^Alcohol Research and Treatment Clinic, Centre for Addiction and Mental Health, Toronto, ON, Canada; ^11^Department of Family and Community Medicine, University of Toronto, Toronto, ON, Canada

**Keywords:** insula, addiction, brain stimulation, transcranial magnetic stimulation (TMS), transcranial direct current stimulation (tDCS), deep brain stimulation (DBS)

## Abstract

Substance use disorders (SUDs) are a growing public health concern with only a limited number of approved treatments. However, even approved treatments are subject to limited efficacy with high long-term relapse rates. Current treatment approaches are typically a combination of pharmacotherapies and behavioral counselling. Growing evidence and technological advances suggest the potential of brain stimulation techniques for the treatment of SUDs. There are three main brain stimulation techniques that are outlined in this review: transcranial magnetic stimulation (TMS), transcranial direct current stimulation (tDCS), and deep brain stimulation (DBS). The insula, a region of the cerebral cortex, is known to be involved in critical aspects underlying SUDs, such as interoception, decision making, anxiety, pain perception, cognition, mood, threat recognition, and conscious urges. This review focuses on both the preclinical and clinical evidence demonstrating the role of the insula in addiction, thereby demonstrating its promise as a target for brain stimulation. Future research should evaluate the optimal parameters for brain stimulation of the insula, through the use of relevant biomarkers and clinical outcomes for SUDs.

## Introduction

Substance use disorders (SUDs) are chronic relapsing brain disorders, characterized by seeking a drug where use becomes compulsive or difficult to control, despite harmful consequences (American Psychiatric Association, 2013). They are complex diseases, characterized by a cycle of intake, withdrawal, craving, and relapse. As a result of drug use and abuse, different neuronal circuits, such as the circuits involved in reward and fear processing, are co-opted to each stage of the addictive cycle and continue to function to maintain addiction (Naqvi et al., 2014; Koob and Volkow, 2016; Chawla and Garrison, 2018). Despite the enormous efforts to find an effective treatment, there are only a handful of approved treatments for these disorders, with limited efficacy as demonstrated by high long-term relapse rates (O’Brien, 2008; Chiamulera et al., 2017).

Given considerable growth in understanding the brain mechanisms underlying addiction, there is an obvious potential to target specific neural regions with brain stimulation techniques. This has fueled a mounting body of research supporting brain stimulation’s efficacy and safety in manipulating underlying neuronal processes and reducing addictive behaviors (for recent reviews see Coles et al., 2018; Hanlon et al., 2018b)^1^. To date, the majority of brain stimulation studies have targeted the dorsolateral prefrontal cortex (dlPFC) (Coles et al., 2018; Hanlon et al., 2018b).

However, in recent years, there is a better understanding of the insula’s critical role in addiction and technological advances enabling stimulation of deeper brain structures (such as the insula) through non-invasive modalities. In fact, up until 2007, the role of the insular cortex in addiction was largely overlooked. In a seminal work, Naqvi et al. (2007) showed that insular damage was correlated with quitting smoking successfully, easily, and without relapse. As a result, the insula (also known as insular cortex, Island of Reil, and insular lobe) became a subject of interest for research on novel addictions treatments.

In this paper we describe brain stimulation techniques that could target the insula and review the insula and its addiction-related functions through an examination of both preclinical and clinical evidence. We also discuss possible limitations of the body of knowledge, as well as future directions.

## Overview of Brain Stimulation Techniques

In this section we discuss three main brain stimulation techniques: transcranial magnetic stimulation (TMS), transcranial direct current stimulation (tDCS), and deep brain stimulation (DBS). Although they are each unique in many ways, they share the principle idea of attempting to induce a behavioral change by stimulating a brain region *via* primary and secondary activations. The effect produced by the stimulation is mainly dependent on the parameters used, which will be briefly discussed below.

### Transcranial Magnetic Stimulation

TMS is a non-invasive brain stimulation technique used to alter magnetic fields to generate electrical currents in a targeted brain area (Barker et al., 1985). The basis of this technique stems from Faraday’s law of induction which states that a time-varying magnetic field drives an electric field of the same magnitude (Daskalakis et al., 2002; Rossi et al., 2009; Lefaucheur et al., 2014). There are many variations of TMS, but the fundamental idea is that of a magnetic coil placed over the scalp, whereby the discharge passes through the scalp, skull, and meninges (unattenuated), allowing the facilitation or suppression of neurons located beneath the coil (Barker et al., 1985; Lefaucheur et al., 2014; Diana et al., 2017).

The first generation of magnetic coils was round coils; they were powerful and easy to use, but less focal (Barker et al., 1985; Deng et al., 2013). Now, there are a number of different coil configurations, such as the figure-8 coil, and more recently, the H-coil. The figure-8 coil, which is the most commonly studied coil, consists of two round coils attached together, leading it to be more focal, yet it can only reach superficial areas (Ueno et al., 1988; Feil and Zangen, 2010), whereas the H-coil is able to reach deeper brain areas (Zangen et al., 2005), such as the insula, though it is less focal.

The parameters that are used during the administration of TMS are of great importance because they can produce differential effects. These settings include the frequency of stimulation, the intensity, the number and type of pulses, and the area being stimulated. The frequency affects the cortical excitability; low-frequency TMS (usually 1 Hz) is generally shown to inhibit cortical excitability, whereas high-frequency TMS (5–20 Hz) is shown to enhance cortical excitability (Gorelick et al., 2014). However, there is debate on whether or not that is always the case (de Jesus et al., 2014), and it seems that the baseline cortical activation state modulates the effect of the magnetic stimulation (Silvanto and Pascual-Leone, 2008). One can administer a single pulse, a paired pulse or if longer lasting changes are required several pulses can be delivered using repetitive TMS (rTMS). One form of rTMS is theta burst stimulation (TBS), whereby pulses are delivered in bursts of three at a frequency of 50 Hz and repeated every 200 ms (Huang et al., 2005). An advantage of this technique is that a session only lasts a few minutes.

As previously mentioned, TMS is a non-invasive form of brain stimulation. Treatment is usually well tolerated and presents no serious side effects. The most common side effects reported are transient headaches and local pain. More serious side effects, most notably seizures, are extremely rare and are unlikely when the proper safety guidelines are followed (Rossi et al., 2009).

### Transcranial Direct Current Stimulation

Transcranial direct current stimulation is another non-invasive brain stimulation technique. However, unlike TMS, tDCS emits a weak current (usually 1–2 mA) *via* two or more electrodes in direct contact with the scalp (Priori et al., 1998; Nitsche and Paulus, 2000). tDCS does not directly result in neuron firing as it does not produce action potentials. Rather, it produces a subthreshold change in neuronal membrane potentials (Purpura and McMurtry, 1965; Nitsche et al., 2008). This brain stimulation technique is not able to reach deep brain areas; however, secondary activations are possible (see Secondary Activation below).

Similar to TMS, parameters can be adjusted to elicit different responses. For instance, the size and placement of the electrodes affect the site being stimulated, current distribution, and focality (Nitsche et al., 2007). Typically, two electrodes are placed on the scalp, one anode and one cathode. The current passes from one electrode, through the skull and through the brain, and then reaches the second electrode (Nitsche et al., 2008). Anodal stimulation usually results in cortical excitability, whereas cathodal stimulation usually results in cortical inhibition (Nitsche et al., 2008). The duration of stimulation is also an important factor; it plays a role in the determination of the aftereffects (i.e., the continued effects after stimulation has stopped). It has been shown that to produce aftereffects of 1 h or more, there needs to be stimulation for at least 9 min (Nitsche and Paulus, 2001). It should be noted that longer or more intense stimulation does not always equate to increased effects. In fact, it can result in an excitatory effect becoming inhibitory and vice versa (Batsikadze et al., 2013). tDCS outcomes have also been shown to be subject to inter-individual variability, possibly due to a number of factors (Wiethoff et al., 2014), one of them being anatomical brain differences (Kim et al., 2014).

Side effects from tDCS are typically mild, such as tingling sensation and light itching under the electrode, and usually dissipate after stimulation. Afterward headaches and, occasionally, nausea are reported. In general, tDCS is well tolerated and presents no serious side effects (Matsumoto and Ugawa, 2016).

### Deep Brain Stimulation

Deep brain stimulation is an invasive brain stimulation technique that requires neurosurgical implantation of bipolar electrodes attached to a permanently implanted pulse generator (usually subcutaneously in the chest) (Greenberg and Rezai, 2014). This technique has the ability to reach deep brain regions, more than any other stimulation technique, and it is highly focal. Currently, DBS is mainly used for movement disorders such as Parkinson’s disease (Kringelbach et al., 2007); however, it has been approved by the U.S. FDA for treatment-resistant obsessive-compulsive disorder. The exact mechanism of DBS is not completely understood, it seems to elicit multiple mechanisms of action (see review by Herrington et al., 2016).

As described for the two previous stimulation techniques, the parameters used can determine to a large extent the neurophysiological response. These include the brain region targeted (to the degree of the precise location in which the electrode is implanted on the target region), intensity and frequency of stimulation, and pulse width (i.e., length of pulses). The combination of all these parameters leads to different outcomes of stimulation. The parameters used are decided on the basis of the treatment intended and can be changed and modified by external programmer devices (Kringelbach et al., 2007).

Although DBS has the capacity to target deeper areas in the brain that might be needed for addiction-related treatments, it should be noted that the risk of adverse events is much higher with this technique. Other than the adverse events that can occur from brain stimulation, this technique requires surgery, hence the added risks that can occur. Although not as common as when DBS first came into practice, hardware failure can still occur (Breit et al., 2004).

## Structural and Connectivity Overview of the Insula

**Figure 1:**
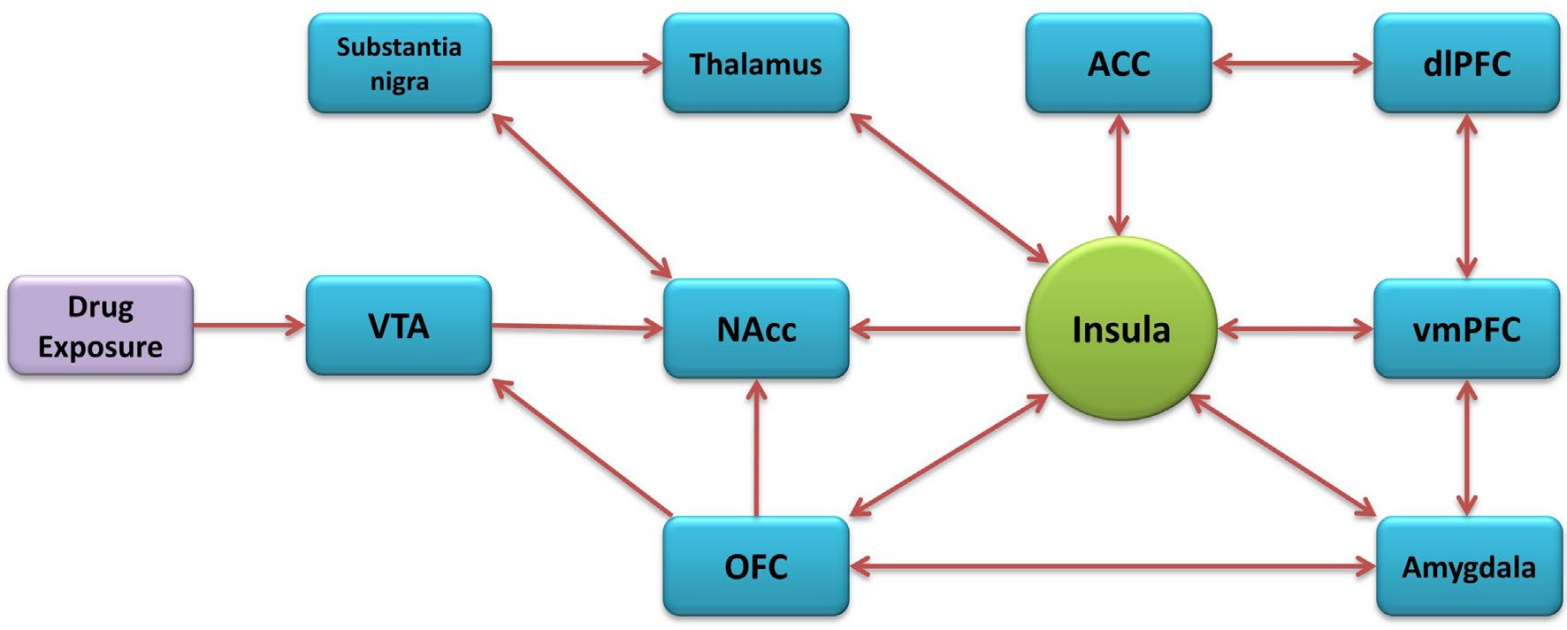
Addiction-related neural circuitry of the insula.

The insular cortex is part of the cerebral cortex, folded in the lateral sulcus. It is commonly divided into anterior and posterior parts, based on the cytoarchitectonic and functional connectivity (Mesulam and Mufson, 1982; Deen et al., 2011; Kelly et al., 2012). It is also composed of three distinct subregions known as the granular, dysgranular, and agranular (Paxinos and Watson, 1986). The insula has major bidirectional connections with the orbitofrontal cortex (OFC), anterior cingulate cortex (ACC), supplementary motor areas, parietal (primary and secondary somatosensory areas), and temporal cortices and with subcortical structures that include the amygdala, globus pallidus, and thalamus. The anterior insular cortex (aIC) sends projections to the caudate-putamen, nucleus accumbens (NAcc), and extended amygdala (McGeorge and Faull, 1989; Wright and Groenewegen, 1996; Shi and Cassell, 1998b; McDonald et al., 1999) while having reciprocal connections with the basolateral amygdala (BLA) and the prelimbic cortex (Gerfen and Clavier, 1979; Saper, 1982; Vertes, 2004). The aIC receives inputs from the medial subdivision of the mediodorsal thalamic nucleus and other various medial thalamic nuclei involved in motivational/affective aspects of nocioception (Krettek and Price, 1977; Van der Werf et al., 2002). The posterior insular cortex (pIC) receives general viscerosensory unimodal inputs (Cechetto and Saper, 1987), nociceptive thalamic inputs (Gauriau and Bernard, 2004) and somatosensory cortex inputs. The pIC also sends projections to the caudate-putamen, thalamus, and somatosensory cortex (Shi and Cassell, 1998a). In general, the aIC has greater connectivity with the frontal lobe, and the pIC has greater connectivity with the parietal lobe (Gasquoine, 2014). Many of the insula’s connectivity constitute areas known to be involved in addiction (**Figure 1**) (Naqvi and Bechara, 2010; Naqvi et al., 2014; Koob and Volkow, 2016).

Dopaminergic neuron terminals of the insula originating in the ventral tegmental area (VTA) and substantia nigra are believed to influence dopamine neuronal reactivity and dopamine release in the NAcc, critical for addictive behavior (Kalivas and Volkow, 2005). The entire granular subregion has notably high dopamine utilization (Gaspar et al., 1989) with the aIC having high density D1-subtype receptors surrounding dopaminergic terminals from the VTA and substantia nigra. These terminals project to the agranular subregion containing large pyramidal neurons with gamma-Aminobutyric acid (GABA) B-subtype receptors (Margeta-Mitrovic et al., 1999) which project to the amygdala and NAcc (Ohara et al., 2003).

Corticotropin-releasing factor (CRF) plays a role in the motivation to consume drugs in human subjects with substance dependence and distress during withdrawal and stress-induced relapse (Contoreggi et al., 2003). The agranular subregion contains a high density of CRF subtype 1 receptors (Sánchez et al., 1999). Furthermore, the insula has a high level of endogenous opioids (Gaspar et al., 1989), high density of µ-subtype opioid receptors (Baumgartner et al., 2006), high density of hypocretin subtype-1 receptors (Hollander et al., 2008), and a high level of nicotinic acetylcholine receptors (nAChRs) containing the β2 subunit (Rubboli et al., 1994), the major subtype of nAChR implicated in nicotine reward (Maskos et al., 2005; Grabus et al., 2006).

## Functional Role of the Insula

The insular cortex is involved in feelings of anxiety, pain, cognition, mood, threat recognition, and conscious urges (Hardy, 1985; Suhara et al., 1992; Craig, 2002; Paulus and Stein, 2006). Although it is involved in a multitude of behaviors, the insular cortex was rarely studied in depth and was believed to be the “primary taste cortex” for decades (Penfield and Faulk, 1955; Pritchard et al., 1999). Since then, several studies have demonstrated the insula’s primary role in sensations of taste and disgust (Ragozzino and Kesner, 1999; Fresquet et al., 2004; Isnard et al., 2004) and the integration of a wide variety of visceral sensations from the airways, gut, and cardiovascular systems (Cechetto and Saper, 1987; Allen et al., 1991), indicating the insula is heavily involved in the integration of internal and external stimuli to guide behavior toward or away from said stimuli for the purposes of maintaining homeostasis.

Studies of epileptic seizures in the insula (Isnard et al., 2004), stroke-induced lesions to the insula (Pritchard et al., 1999), lesioning studies in rats (DeCoteau et al., 1997; Ragozzino and Kesner, 1999; Fresquet et al., 2004), and direct electrical stimulation studies in primates (Verhagen et al., 2004) have shown the insula is critical for perception, recognition, and working memory in taste. The importance of visceral perception in addiction was first noted in the late 1980s, as dopaminergic innervation of the insular cortex was found to play an important role in the conditioning of the aversive aspects of opiate withdrawal (Zito et al., 1988) and the active avoidance of withdrawal. The insular cortex is critical for experiencing disgust and recognizing it in others, causing learning and memory of disgust to play a critical evolutionary role in mammals (Woolley et al., 2015).

Beyond acting as the primary taste cortex, the insula’s critical thalamic connections allow it to integrate a range of visceral sensations, including inputs from the airways, gut, and cardiovascular systems (Cechetto and Saper, 1987), all known to be relevant with regard to bodily responses to the performance of addictive behaviors (Verdejo-Garcia et al., 2007). AD Craig first recognized the insula as the critical brain region underlying “interoception” (Craig, 2002), a process that integrates internal signals and external stimuli to maintain homeostasis (Craig, 2003). The pIC keeps a constant account of the current state of the body; this information is then relayed through the thalamus and dysgranular insular cortex, which appears to integrate salient external stimuli (Craig, 2004). The aIC compares the current state of the body and environment to prior states and environments to maintain homeostasis (Craig, 2009); such influence from representations of all bodily signals, and integration with external stimuli to guide behavior toward or away from said stimuli, indicates the aIC plays a significant role in drug seeking behavior. Through a study measuring skin conductance, anxiety ratings, and blood oxygenation level-dependent (BOLD) functional magnetic resonance imaging (fMRI) were used to assess responses to simulated threats, Alvarez et al. (2015) found that exaggerated insula activation during the threat of unpredictable shock is directly related to low perceived control in anxiety-prone individuals, supporting prior evidence that individuals prone to anxiety may have exaggerated activity in the anterior insula and altered activity in the cingulate cortex during anticipation of aversive events. Interestingly, individuals with methamphetamine dependence have a reduced emotional response to threatening scenes, empathy for another’s pain, and fearful or angry face, all of which correlate with hypoactivation of the insula (Kim et al., 2011).

In addition to important interoceptive and homeostatic functions, the insula plays a major role in all aspects of decision making (Droutman et al., 2015). Bechara and Domasio’s “Somatic Marker Hypothesis (SMH)” theory is one of several that offers an explanation of the insula’s involvement in decision making, postulating that somatic markers of feelings in the body (e.g., rapid heartbeat in response to anxiety) influence decision-making, and highlighted the insula’s role in decision making through its reactivation alongside the ventral medial prefrontral cortex (vmPFC) and amygdala as part of somatic state patterns previously learned by subjects evaluating a familiar stimulus (i.e., interoception) (Bechara and Damasio, 2005). Evidence from electroencephalogram (EEG) studies (Wiese et al., 2005; Mueller et al., 2007) has shown the insula’s activation when “intentional” actions are initiated when faced with multiple external options. Intentional acts result from internal motivation and planning, versus automatic acts that are triggered by external stimuli (Brass and Haggard, 2010).

## Large-Scale Brain Networks in Addiction

Current neuroscience approaches investigate communication between brain regions in addition to the investigation of specific brain areas. fMRI allows exploration of fluctuations of BOLD signals in different brain regions which in turn allow the exploration of internetwork as well as intranetwork connectivity (Menon, 2011; Lu and Stein, 2014). In addictions, the main focus is on three well-established intrinsic networks: the default mode network (DMN) which includes the vmPFC and posterior cingulate cortex (PCC), the executive control network (ECN) hooked in the dlPFC and posterior parietal cortex (PPC), and the salience network (SN) which includes the aIC and ACC. The DMN is active when an individual is not focused on external tasks, and it is related to activities like day dreaming. In the context of addictions, it can be related to rumination about drug use. The ECN is active when an individual is focused on external tasks that demand cognitive functioning (e.g., working memory). The SN dynamically switches between the ECN and DMN, which are generally anticorrelated, following detection of relevant stimulation or condition (Menon and Uddin, 2010).

Sutherland et al. proposed a framework to understand the large-scale brain networks dynamics in addictions. They showed that under nicotine deprivation, the SN would switch to DMN, hence, direct attention resources toward internal withdrawal symptoms, while under nicotine administration, the SN would switch to ECN, hence direct attentional resources toward external stimuli and executive functions (Sutherland et al., 2012; Lerman et al., 2014; Sutherland and Stein, 2018). Therefore, an aberrant function of the SN (including the insula) may lead to an aberrant brain function, such as addiction. The activation of the insula and large-scale brain networks in addicted individuals is further demonstrated in the Neuroimaging Studies section below.

## Preclinical Evidence for the Insula

As the critical brain region underpinning interoception, it is not surprising to see the insula involved in all major aspects of addiction. For simplicity sake, we provide here a short review of the preclinical literature examining the roles of various cytoarchitectural subregions of the insula in animal models representing each stage of the addictive cycle, as proposed by Koob and Volkow (2010): 1) Binge/Intoxication, 2) Withdrawal/Negative Affect, and 3) Preoccupation/Anticipation. The anterior agranular subregion (aAgIC) or posterior granular subregion (pGIC) is typically targeted in rodent models, as specific targeting of the dysgranular subregion is difficult with local infusions.

### Binge/Intoxication Stage

Since the seminal findings of Naqvi and colleagues in human smokers (Naqvi et al., 2007), the majority of preclinical research on the IC’s role in addiction has utilized nicotine. Noncontingent nicotine exposure has been demonstrated to induce a lasting increase in dendritic complexity (length/bifurcations) in the aAgIC (Ehlinger et al., 2012) while noncontingent nicotine pyrrolidine methiodide, which does not penetrate the blood–brain barrier, activates the aAgIC (Dehkordi et al., 2015). The acquisition of nicotine-induced conditioned place preference (CPP) is blocked by lesions to the aIC (Scott and Hiroi, 2011). Our own laboratory demonstrated that aAgIC or pGIC chemical inactivation attenuates nicotine taking, under various ratio schedules of reinforcement (Forget et al., 2010; Pushparaj et al., 2015), with similar attenuation observed when the pGIC was electrically inactivated (Pushparaj et al., 2013). These methods of inactivation demonstrated no effect on food taking in rats under a moderately restricted diet. Similar attenuation of nicotine, but not food, taking has been demonstrated by blockade of hypocretin-1 receptors in the pGIC, which is densely innervated by hypocretin-1 peptide-containing neurons (Hollander et al., 2008). Attenuation of nicotine taking is also observed following blockade dopamine D1, but not D2, receptors in the aAgIC (Kutlu et al., 2013), though high doses of D1 antagonists also attenuate food taking (Di Pietro et al., 2008).

Noncontingent alcohol exposure has been demonstrated to inhibit aIC activity (Jaramillo et al., 2016), which is unsurprising given it produces global cortical inhibition. *In vitro* ethanol inhibits electrically-evoked N-methyl-D-aspartate (NMDA) receptor-mediated excitatory post-synaptic currents (EPSCs) in brain slices of the aAgIC (Shillinglaw et al., 2018). Long-term synaptic depression (LTD) in the dorsolateral striatum also occurs exclusively at inputs from the aIC and is selectively disrupted by *in vivo* alcohol exposure (Muñoz et al., 2018). Inactivation of the aAgIC (Jaramillo et al., 2016) or chemogenic silencing of the aIC projections to the nucleus accumbens core (NAccCore) (Jaramillo et al., 2017), both produce sensitivity to the interoceptive effects of alcohol. Optogenetic inhibition of aIC glutamatergic input to the NAccCore attenuates aversion-resistant alcohol taking, but not alcohol taking in the absence of aversive stimuli (Seif et al., 2013). Contrarily, a recent study utilizing chemogenic partial inhibition demonstrated an increase in alcohol taking when the aIC was inhibited, but an attenuation in alcohol taking when aIC projections to the NAccCore were silenced, with the latter having no effect on sucrose taking (Jaramillo et al., 2018a; Jaramillo et al., 2018b). Our own laboratory has demonstrated that inactivation of the pGIC produces an attenuation of alcohol taking (Pushparaj and Le Foll, 2015).

Contrarily, aAgIC lesions have no effect on the acquisition of cocaine taking (Pelloux et al., 2013), nor does inactivation of the aAgIC affect established cocaine-taking (Pietro et al., 2006). In fact, lesions facilitate the escalation of cocaine intake, yet post-escalation lesions still reduce intake (Rotge et al., 2017). Interestingly, extended access self-administration, but not noncontingent exposure, to cocaine has also demonstrated effects on aAgIC-dependent learning (Kantak et al., 2005) and self-administration has also been shown to reduce glucose utilization in the aAgIC in primates (Porrino et al., 2002). Blockade of dopamine D1 receptors in the aAgIC does reduce cocaine intake; however, it also disrupts food intake, suggesting a global disruption of behavior (Di Pietro et al., 2008).

Finally, inactivation of the pGIC, but not the aAgIC, prevents the acquisition of CPP induced by morphine, without producing any effect on general motor and/or spatial learning (Li et al., 2013). Lesions of the aIC or pIC both produce no effect on the acquisition of morphine-induced CPP (Mackey et al., 1986; Sun et al., 2018).

Though the IC appears to play a greater role in the binge/intoxication stage for some addictive drugs (e.g., nicotine, alcohol) over others (e.g., cocaine), the region does appear to play some role in the neurocircuitry underlying this stage of addiction for all addictive drugs examined.

### Withdrawal/Negative Affect

Inactivation of the aAgIC or pGIC prevents the acquisition of conditioned place aversion (CPA) induced acute morphine withdrawal (Li et al., 2013). Similarly, lesions of the aIC prevent the acquisition of CPA from morphine withdrawal (Wang et al., 2016). There is greater activation of glutamatergic neurons and signaling protein expression in the medial IC during protracted withdrawal, as compared to acute withdrawal from morphine (Ma et al., 2018). The anxiety-like behavior observed during protracted withdrawal is alleviated by lesions of the medial IC. The cannabinoid system has also been implicated, with the inhibition of monoacylglycerol lipase (causing elevation of the endocannabinoid 2-arachidonyl glycerol; 2-AG) in the pGIC preventing the acquisition of CPA from morphine withdrawal, which was reversed by the blockade of cannabinoid-1 receptors (Wills et al., 2016).

Contrary to the findings with morphine, lesions of the aIC had no effect on the acquisition of CPA induced by acute nicotine withdrawal (Scott and Hiroi, 2011). Though the studies on morphine withdrawal strongly support a critical role of the IC in the neurocircuitry underlying this stage of addiction, further work should be conducted to explore the role of the IC in other addictive drugs for which withdrawal is a major barrier to abstinence (e.g., alcohol).

### Preoccupation/Anticipation

Our own laboratory demonstrated that aAgIC or pGIC inactivation attenuates nicotine seeking (Forget et al., 2010; Pushparaj et al., 2015), with similar attenuation observed when the pGIC was electrically inactivated (Pushparaj et al., 2013). The incubation of nicotine seeking (i.e., greater seeking after 7 days vs. 1 day of abstinence) is associated with higher levels of dopamine (DA)- and cyclic adenosine monophosphate (cAMP)-regulated phosphoprotein of 32 kDa (DARPP-32), enriched in DA neurons containing DA D1 receptors, in both the aAgIC and pGIC (Abdolahi et al., 2010).

Inactivation of the aAgIC, but not the pGIC, attenuates cue-induced cocaine seeking, while having no effect on drug priming-induced cocaine seeking or food seeking induced by either cues and/or priming (Cosme et al., 2015). Corticotropin-releasing factor (CRF) receptor-1 blockade of the aAgIC was also found to attenuate cue-induced cocaine seeking. Inactivation of the aAgIC attenuates context-induced reinstatement of cocaine seeking without altering locomotor activity; however, inactivation immediately following re-exposure to drug context has no effect on memory reconsolidation (Arguello et al., 2017). Interestingly, lidocaine inactivation of the aAgIC attenuates cue+context-induced cocaine seeking when the context was established by odor but not by sound (Pietro et al., 2006), which may be because gustation is influenced by odor, with the aAgIC being part of the gustatory cortex (Pritchard et al., 1999). In contrast to these studies, one study demonstrated an increase in cocaine seeking in aAgIC-lesioned rats (Pelloux et al., 2013); however, that effect is likely due to the rats having been lesioned prior to the acquisition of cocaine taking, as lesions post-acquisition result in an attenuation of drug-induced cocaine seeking (Rotge et al., 2017).

The presentation of amphetamine-induced CPP activates the pGIC and inactivation of the subregion prevents the presentation of said place preference (Contreras et al., 2007). Subsequent work from the same research group demonstrated a similar effect on the presentation of CPP when the protein synthesis inhibitor anisomycin was infused into the pGIC immediately following reactivation of CPP, and that this effect was longer lasting when anisomycin was infused into the higher-order aAgIC (Contreras et al., 2012). *In vivo* microdialysis of the aAgIC has also shown decreased depolarization-evoked GABA release in rats previously chronically exposed to methamphetamine (Mizoguchi et al., 2015). Inactivation of the aIC is capable of attenuating relapse to methamphetamine taking following the removal of contingency management (i.e., palatable food pellets mutually-exclusive from drug) (Venniro et al., 2017). The presentation of morphine-induced CPP is also abolished by lesions to the pIC (Sun et al., 2018) or the inhibition of nitric oxide in the pIC (Ma et al., 2014).

The IC appears to play various roles in the neurocircuitry underlying this stage of addiction, though differentially depending on the addictive drugs examined and the type of intervention (i.e., types of inactivation, lesions, etc.). Overall, there is evidence of some role for the IC in the neurocircuitry underlying all stages of the addictive cycle, clearly implicating the IC as a fundamental region in the maintenance and relapse to addictive drugs. Though this may seem contradictory to the idea of the IC as simply a part of the salience detection system, it is not unreasonable to posit that its interoceptive functions should play some role in drug intake, withdrawal, and anticipation. Future work should continue to evaluate the differential roles of the aAgIC and pGIC in various models of the stages of addiction.

## Clinical Evidence for the Insula

In this section, we provide the clinical evidence implicating the insula in addiction. Unlike the preclinical evidence which distinguishes between the addiction stages, clinical work does not always do so. For that reason, we present the clinical data based on study design: lesion, neuroimaging, and brain stimulation.

### Lesion Studies

Despite the fact that the relevance of the insula for addiction had been observed in studies before, Naqvi et al. (2007) brought attention to its fundamental role in addiction. They found that smokers with damage to the insula experienced disruption of smoking addiction more so than those with damage not involving the insula. These smokers were able to quit smoking easily, immediately after damage onset, without relapse, and without any urge to smoke since quitting (Naqvi et al., 2007). Since then, other lesion studies have emerged, indicating that lesion to the insula best predicts smoking cessation and the probability of quitting smoking is five folds greater than when there is not damage to the insula (Suñer-Soler et al., 2012). Although, insular damage is associated with increased odds of smoking cessation, those that do relapse tend to remain abstinent for longer periods than those with non-insular damage (Abdolahi et al., 2015a). It has also been shown that smokers with insular damage experience less frequent and severe withdrawal symptoms of nicotine (Abdolahi et al., 2015b) and less smoking urges (Abdolahi et al., 2017). Also, damage to the insula was associated with complete abstinence from all nicotine products (Abdolahi et al., 2015a). Furthermore, damage to the basal ganglia was found to significantly increase rates of smoking cessation compared to damage elsewhere (Gaznick et al., 2014). However, when the damage included both the basal ganglia and the insula, it resulted in even higher rates of smoking cessation (Gaznick et al., 2014). Thereby supporting the notion of the insula being a key player in addiction. Nonetheless, one study found no relation between insula damage and smoking status (Bienkowski et al., 2010). It was later suggested that this disparity could be due to the fact that the study took place in Poland, where the harm of smoking is not widely perceived (Naqvi et al., 2014).

Although the vast majority of the evidence pertains to nicotine, other addictions have been shown to be affected by insular damage. For instance, in a comparison between ischemic stroke patients who used opium prior to their stroke, patients with insular damage were significantly more likely to quit using opium; this effect was more significant among younger patients (Yousefzadeh-Fard et al., 2013). Insular lesions were also correlated with the abolishment of cognitive distortions during gambling simulations compared to healthy controls and patients with lesions to other brain regions. This is of importance as problem gamblers are at greater risk of having the examined cognitive distortions such as near misses—a loss that falls close to a jackpot and gambler’s fallacy—distorted psychological processing of random sequences (Clark et al., 2014).

Altogether lesion studies show that insular damage improves nicotine and opium cessation outcomes in patients suffering from strokes. Future research should explore if that is also the case in other SUDs.

### Neuroimaging Studies

#### Structural Changes

There is a considerable amount of evidence from structural imaging studies of the insula in addicted individuals. In smokers, researchers have found that cortical thickness and gray matter density of the insula is significantly decreased compared to non-smokers (Fritz et al., 2014; Li et al., 2015; Hanlon et al., 2016; Stoeckel et al., 2016). Furthermore, a meta-analysis of 15 papers (761 smokers and 1,182 non-smokers) found that smoking was associated with decreased gray matter in the insula, specifically the left insular cortex (Sutherland et al., 2016). Although the majority seem to suggest a decrease in cortical thickness and gray matter density, one study found the opposite effect, such that smokers had increased gray matter densities (Zhang et al., 2011). Also, it appears that the changes in insular cortex density are more pronounced in older, more established, smokers as opposed to younger smokers (Hanlon et al., 2016). Young smokers (aged 16–21) did not differ in insula thickness compared to non-smokers (Morales et al., 2014). This suggests that the structural changes could be due to prolonged exposure to smoking. In terms of correlations with smoking behaviors and markers, there is evidence showing that gray matter density of the insula is negatively correlated with daily cigarettes smoked (Stoeckel et al., 2016), Fagerström Test for Nicotine Dependence (FTND) scores (Wang et al., 2019), and pack year smoking (Morales et al., 2014). Smoking cessation outcomes in relation to structural changes of the insula have also been evaluated, but showed no significant difference between smokers who quit, smokers who relapsed and non-smokers (Wang et al., 2019).

Nicotine is not the only drug that has been shown to cause structural changes to the insula; changes have been reported in cocaine, heroin, methamphetamine, cannabis, and alcohol use. For instance, cocaine dependence and duration of dependence has been shown to be correlated with decreased gray matter volume of the insula, which in turn was associated with greater impulsivity (Ersche et al., 2011). Lower cortical thickness of the insula was also found in cocaine users compared to healthy controls (Geng et al., 2017). In addition, cocaine- and heroin-dependent individuals had lower gray matter values in the right pIC compared to healthy controls (Gardini and Venneri, 2011). A meta-analysis of structural changes among stimulant-dependent individuals revealed significant decreases in gray matter in the left insula (Ersche et al., 2013). Similarly, another meta-analysis found cocaine and methamphetamine users had gray matter reductions in the insula. The right insula was more affected in cocaine users, whereas for methamphetamine users it was the left insula. Also, longer methamphetamine abstinence was correlated with an increase in gray matter of the left insula. Interestingly, the duration of methamphetamine use was associated with increased gray matter in the right insula (Hall et al., 2015). With regard to cannabis, regular smokers compared with occasional smokers were found to have a reduction in gray matter volume of the left insula, and this was correlated with the frequency of cannabis use in the 3 months prior to study entry (Battistella et al., 2014). Alcohol use has been subject to both white and gray matter changes. Adolescents with alcohol use disorder had greater white matter volume in the left insula, which was correlated with enhancement motives (i.e., the feeling of “being high”) for drinking and with the frequency of binge drinking at baseline and 1-year follow-up. Furthermore, greater volume of white matter in the right insula was correlated with alcohol obsession/craving (Chung and Clark, 2014). In terms of gray matter, adolescents who drank alcohol excessively, but did not meet the criteria for alcohol use disorder, had lower volumes in the right insula compared to light drinkers (Heikkinen et al., 2017).

Behavioral addictions have also been studied. Individuals with the inability to control Internet use even when accompanied by negative consequences to their life showed significantly lower gray matter density in the left insula and lower white matter density in the right insula (Lin et al., 2015). Individuals with online gaming addiction showed gray matter atrophy in the insula, which was correlated with the severity of the subjects’ addiction (Weng et al., 2013). A study among subjects who use social media excessively found a correlation between the severity of addiction-like symptoms and a reduction of gray matter volumes in the bilateral pIC, as well as impulsivity (Turel et al., 2018).

Altogether the evidence suggests that structural changes to the insula occur in addicted individuals, thus further demonstrating its involvement in addiction. Although there is not complete consensus on what the changes are, the majority of the findings point toward a decrease in gray matter volumes and density of the insula, rather than an increase.

#### Functional Changes

##### Resting State Functional Connectivity

Resting state functional connectivity (rsFC) is a method used to measure brain activity between regions while in a resting state (i.e., task-negative state) (Biswal et al., 1995). It has been widely used in addiction studies to investigate the neurocircuitry, such as large-scale brain networks discussed above, that underlie addiction. For instance, decreased rsFC between the right aIC (component of the SN) and right superior frontal gyrus (component of the ECN) has been reported in smokers (Fedota et al., 2016). Furthermore, smokers were found to have lower rsFC between the insula, executive functions regions, such as the OFC, and superior frontal gyrus, whereas the rsFC between the insula and anterior ACC seems to be negatively correlated with FTND scores (Zhou et al., 2017). Abstaining from smoking for just 12 h resulted in increased rsFC between the right aIC and executive functions regions (i.e., right OFC, and vmPFC) as well as the interconnectivity in the SN (Bi et al., 2017). Acutely abstinent smokers also demonstrate increased rsFC of the insula-DMN when compared to both non-smokers (Huang et al., 2014) and satiated smokers, though satiated smokers still have higher insula-DMN rsFC than non-smokers. Furthermore, a study comparing BOLD data of smokers before and after 48 h of abstinence found that the overall dynamics rsFC was decreased during abstinence and that dorsal and posterior insular connections to the DMN and SN were enhanced, whereas the ventral insular connection to the ECN was reduced. Also, static rsFC in the ventral and posterior insular-seeded circuits were correlated with subjective ratings of aversive affect and withdrawal symptoms (Fedota et al., 2018). However, one study found that increased risky decision making in smokers, which is associated with stronger nicotine dependence, resulted in increased rsFC between the aIC and dorsal ACC (dACC) and the right and left aIC (Wei et al., 2016). A meta-analysis of pharmacological neuroimaging studies found that among smokers, both administration of nicotinic acetylcholine receptor agonists and cigarette smoking were associated with decreased activity in the regions associated with the DMN and SN, such as the insula, vmPFC, PCC, and increased activity in the regions associated with the ECN, such as the lateral PFC and dACC (Sutherland et al., 2015). Interestingly, smokers with higher alexithymia appear to have lower levels of increased rsFC between the right aIC and the vmPFC, a core node of the DMN, and that this mediates craving following overnight withdrawal, though this mediation is disrupted by varenicline or nicotine replacement therapy (Sutherland et al., 2013b). A group of researchers have investigated the functional connectivity of smokers and non-smokers with regard to *CYP2A6* genetic variation. *CYP2A6* variation affects the rate of nicotine metabolism and is associated with smoking outcomes. In smokers, it was found that slow metabolizers of *CYP2A6* had reduced functional connectivity strength in the dACC and ventral striatum, which was driven by the bilateral insula (Li et al., 2017a).

In terms of other SUDs, individuals with cocaine use disorder had greater connectivity within the SN and weaker connectivity between the SN and the striatum. The reduced connectivity was correlated with impulsivity in cocaine users, and can be conceptualize as reduced inhibition of the SN on the striatum. Interestingly, the aberrant connectivity was not correlated to current craving (Wisner et al., 2013). Other researchers have found cocaine users to have decreased connectivity between the right and the left insula, as well as to the dACC, thus affecting the SN (Geng et al., 2017). Similarly, alcohol users had lower SN connectivity than controls and an attenuated blood flow to the insula (Sullivan et al., 2013), whereas individuals with heroin use disorder had an increased SN connectivity (Wang et al., 2010). Reduced connectivity between the aINS and the dACC in regular binge drinkers was found when given alcohol compared to placebo. In addition, the more the connection was reduced, the calmer the participants reported feeling, thus suggesting that the decreased connectivity between these two regions of the SN may be linked to the rewarding effects of alcohol (Gorka et al., 2018). For behavioral addictions, internet gaming disorder subjects exhibited enhanced rsFC between the aIC and a network of regions including the ACC, putamen, angular gyrus, and precuneus; and between the pIC and postcentral gyrus, precentral gyrus, supplemental motor area, and superior temporal gyrus (STG). The addiction severity was positively associated with connectivity between the aIC and angular gyrus, and STG, and with connectivity between the pIC and STG (Hanlon et al., 2018a). Another study found that internet gaming disorder individuals showed decreased functional connectivity between the left pIC, bilateral supplementary motor area and middle cingulated cortex, and between the right pIC and right superior frontal gyrus, and decreased functional integration between insular subregions. This finding can be conceptualized as a reduction in the ability to inhibit motor responses to internet games (Zhang et al., 2016).

In summary, the involvement of the insula in addiction was demonstrated in many rsFC studies. Alterations in the SN function and in the connectivity of the insula to executive functions regions were seen. This may be interpreted as the SN impaired ability to switch from cognitive states of interoceptive cravings to cognitive control. However, more studies are needed to gain a better understanding of brain networks in addictions.

##### Cue Reactivity

Non-resting state studies have also demonstrated altered connectivity in smokers. Smokers who participated in a memory retrieval task involving smoking and neutral cues were found to have the left insula significantly more active during the retrieval of smoking cues compared to neutral cues. Smokers also reported more cravings when viewing smoking cue images (Janes et al., 2015). Smoking cues resulted in greater left insula functional connectivity with the right insula, OFC, and striatum with that greater connectivity being positively correlated with nicotine dependence, as judged by scores on the FTND (Claus et al., 2013). There was also increased activation in the right aIC and striatal regions in response to cues relating to rewards or losses when nicotine was administered compared to placebo (Moran et al., 2018). Similarly, craving in smokers when viewing smoking cues was associated with increased connectivity, but between the right aIC and the precuneus, which are two regions known to play a role in interoception and self-awareness (Moran-Santa Maria et al., 2015). A meta-analysis of fMRI studies on smoking cue reactivity also concluded that smoking cues, as compared to neutral cues, reliably evoke larger fMRI responses in the insula (Engelmann et al., 2012). The effect of smoking initiation-related cues (e.g., lighter) vs. smoking conclusion-related cues (e.g., cigarette butt in ashtray) on two groups of smokers who were either content or discontent with their behavior further examined temporal relations of smoking cues (Stippekohl et al., 2012), with discontent smokers showed greater insular activation in response to initiation-related cues than content smokers, and aIC activation is observed during intrinsic, but not extrinsic, motivation (Lee and Reeve, 2013).

A study that was done on substance-dependent individuals who used cocaine, alcohol, or nicotine demonstrated an increased BOLD signal to substance cues relative to neutral cues in three distinct clusters: the medial PFC/ACC, the left inferior frontal gyrus/insula, and the right premotor cortex (Hanlon et al., 2018a). This finding may suggest different brain involvement among different individuals with SUD. Interestingly, the three distinct clusters were seen in cocaine, alcohol, or nicotine abuser and, therefore, are not related to the drug of choice. Abstained cannabis users demonstrated greater activation in response to the cannabis cues as compared with the neutral cues in several structures in the reward pathway, including the insula, VTA, thalamus, ACC, and amygdala (Filbey et al., 2009). Surprisingly, a 2011 meta-analysis did not support the role of the insula in drug cue activity- or craving-related activity. However, this might be due to the meta-analysis rigid exclusion criteria (Chase et al., 2011).

In summary, there is evidence suggesting the involvement of the insula in reactivity to drug cues. However, further investigation, such as a meta-analysis, might be of great value. It is important to note that there is debate in the literature about the way drug cues reactivity and craving influence relapse risk, thus additional research should also investigate this question (for further reading on this subject see review by Garavan, 2010).

##### A Tool for Prognosis and Relapse Prediction

A better understanding of rcFC and cue reactivity led the way to try to find a tool that will enable better prognosis assessment and relapse prediction. In smokers, researchers found a decreased functional connectivity between the insula and the primary sensorimotor cortices (Addicott et al., 2015), as well as the insula, dACC, and dlPFC (Janes et al., 2010), in those who relapsed. Also, abstinent smokers had greater activation in the aIC during an inhibitory control task. Therefore decreased insula activation leads to decreased inhibitory control, which could be an indicator of relapse (Gilman et al., 2018). However, individuals who relapsed had greater brain reactivity to smoking cues in the right insula and dorsal putamen (Janes et al., 2017). Sutherland and Stein suggested that neurocircuits centered in the insula may represent a biomarker for acute withdrawal (Sutherland and Stein, 2018). This was demonstrated by rsFC alterations where abstinent smokers had greater connectivity between the insula and the amygdala (a connectivity which underlines negative affect) (Koob and Le Moal, 2005; Koob and Volkow, 2010), but was down-regulated with nicotine or varenicline administration (Sutherland et al., 2013a).

Additionally, abstinent methamphetamine-dependent individuals who relapsed demonstrated attenuated bilateral insula, bilateral striatum, left inferior frontal gyrus, and left ACC activation in response to outcomes of winning, tying, and losing feedback in a reinforcement learning task, compared to those who remained abstinent (Stewart et al., 2014). Methamphetamine users who remained abstinent showed increased activation of the left aIC and right striatum for large/risky relative to small/safe rewards, whereas individuals who relapsed did not show differential activation between reward types (Gowin et al., 2015). Regarding relapsed cocaine-addicted individuals, they displayed reduced connectivity between the bilateral putamen and pIC, which partially mediates higher impulsivity (Battistella et al., 2014). Researchers have found that occasional stimulant users (amphetamine and cocaine) that developed stimulant use disorders had lower activation of the insula and ACC at baseline during a decision making task compared to individuals who stopped using stimulants (Stewart et al., 2017). Heavy alcohol drinkers had an increased BOLD fMRI signal in response to alcohol cues in the insula (it was significant in the left insula and a trend in the right) and the ventral striatum compared to light drinkers. This links the insula and dorsal striatum to relapse vulnerability. In addition, heavy drinkers had reduced responses in frontal areas to pictures related to higher-order life goals and in the cingulate cortex to appetitive food stimuli, suggesting that they have difficulty finding alternative goals. By using this activation pattern it was possible to differentiate between heavy and light drinkers to a high degree of precision (Ihssen et al., 2011).

In summary, it has been suggested in several studies that insula-related activation patterns can be used as a prognostic tool, as well as the link between the insula and striatum, which might suggest that an adequate striatal inhibition by the insula might be a good prognostic factor. The use of the insula as a biomarker is worth further investigation.

### Brain Stimulation Studies

Brain stimulation studies for addiction have mainly targeted the PFC, specifically the dlPFC (Salling and Martinez, 2016). Studies targeting the insula are lacking as there is currently only one study completed thus far (Dinur-Klein et al., 2014). Smokers (*n* = 115) were randomized to either receive 13 sessions of high frequency, low frequency or sham stimulation to the lateral PFC and insula bilaterally. This stimulation was done using an H-coil for deep TMS. High-frequency deep TMS (10 Hz) was found to significantly reduce cigarette consumption, as well as nicotine dependence. Those who also had smoking cues presented during high-frequency stimulation had the best results (44% abstinence at end of treatment and 33% at 6 months follow-up). It should be noted that they did not exclusively target the insula, thus it is not possible to know if the clinical effect were mediated by the insula or the PFC or both. Nonetheless, given the strong evidence of the insula’s role in addiction, as well as the promising results presented by Dinur-Klein et al. (2014), further studies targeting the insula are needed. Currently, there are two smoking cessation trials being conducted using deep rTMS; however, both are targeting the insula along with the PFC (NCT02126124, NCT03264313). On the other hand, our laboratory is working on a clinical trial investigating a combined treatment for smokers using varenicline and deep rTMS targeting the insula. It should be noted that one study found that in healthy volunteers, one session of low-frequency rTMS (1 Hz) to the right aIC using an H-coil was unable to affect the activity of the insula, as measured by behavioral markers (i.e., a blink suppression task and a forced-choice risk-taking task) (Spagnolo et al., 2019).

#### Secondary Activation

It is evident that the target brain region being stimulated is a very important factor that needs careful consideration. However, the primary stimulation may lead to secondary activations. These activations can occur due to neurotransmitter release or from back propagation of action potentials from the primary site of activation (Diana et al., 2017). Furthermore, these activations are not necessarily confined to the networks associated with the primary site, but can be widespread (Gratton et al., 2013). Secondary activations have been demonstrated by studies combining brain stimulation and brain imaging. For instance, one session of high frequency (10 Hz) of rTMS over the left dlPFC in smokers resulted in inhibition of activity in the right insula and thalamus as measured by fractional amplitude of low-frequency fluctuation (fALFF) (Li et al., 2017b). Also, a single session of bilateral tDCS applied over the dlPFC of abstinent methamphetamine users led to a decrease in subjective craving. The subjective feeling was correlated with a modulation of resting state networks; DMN connectivity decreased, whereas ECN and SN intranetwork functional connectivity increased (Shahbabaie et al., 2018). In alcohol users, a single day of 6 trains of continuous TBS to the left frontal pole decreased evoked BOLD signal in left OFC, insula, and lateral sensorimotor cortex without a significant effect on subjective craving (Hanlon et al., 2018a). Interestingly, a publication about one patient with alcohol dependence, reported that a single rTMS to the dACC led to a temporary abolishment of alcohol craving correlated with a decrease in beta EEG activity in the right insula (De Ridder et al., 2011). There has been one study investigating the changes in dopamine levels following bilateral insula rTMS, but it was done on healthy participants. Low-frequency rTMS (1 Hz) was found to significantly decrease dopamine levels in the substantia nigra, sensorimotor striatum, and associative striatum (Malik et al., 2018).

Altogether, these studies suggest that the effect of brain stimulation is not restricted to the area being targeted, but rather can affect a wide variety of areas, which should be considered when deciding on the parameters used for stimulation. In instances where one session of brain stimulation did not yield behavioral change, further investigation should seek to see whether longer courses of stimulation may lead to a behavioral change.

## Discussion

Although there is a vast literature about the use of brain stimulation to treat addictions, there is an ongoing debate about the most effective protocol. Most studies in this field targeted the dlPFC (for recent reviews see Coles et al., 2018; Hanlon et al., 2018b). In this review, we suggested the insula as a novel target to treat addictions. We reviewed brain stimulation techniques and principles that enable stimulation of the insula. In addition, we reviewed relevant preclinical studies which demonstrated the insula’s involvement in all stages of addictions: binge/intoxication, withdrawal/negative affect, and preoccupation/anticipation, as well as the clinical studies which demonstrate the major role of the insula in addictions.

At first glance, the evidence presented may seem to be somewhat contradictory. Structural studies showed that addiction resulted in a loss of gray matter in the insula, thus suggesting a loss of function. On the contrary, neuroimaging studies showed increased activation of the insula, thereby suggesting a gain of function. However, it has been previously suggested that the decrease in gray matter actually means an overactive insula (Droutman et al., 2015; Turel et al., 2018). Naqvi et al. (2014) described that with addiction the insula may undergo both a loss and gain of function depending on the specific function investigated. After drug taking, the insula later plays a role in establishing the internal state feeling of the effects of the drug. This role in interoception is what is believed to lead to continuous drug-seeking behavior, as individuals need to reach homeostatic levels. Therefore, this can be thought of as a gain of function of the insula in individuals with addiction. On the other hand, addiction may cause a loss of function in the insula with regard to risky decision making. There is a decreased capability when it comes to weighing out the negative consequences that can occur from decisions involving drug-seeking, leading to the inability to stop this behavior. This dual effect of addiction on the insula further increases the complexity of this area. However, we can learn from lesion studies that a loss of function improves cessation probability, therefore it might be that the gain of function is the key factor in addictions. However, understanding of the reciprocal relationship of the insula and other brain regions will lead to a better understand in its role in addiction, as the insula should not be thought of in isolation but rather as part of a network.

Although the evidence of the involvement of the insula in addiction is convincing, there is a lack of research translating this into treatment for addicted individuals. No studies have yet to investigate brain stimulation of the insula in addiction, with the exception of one clinical trial in smokers that targeted the insula and PFC together (Dinur-Klein et al., 2014). Since the results of the trial were positive and the increasing evidence of the functional role of the insula, the next course of action should be to see whether stimulating the insula exclusively or with other brain regions posits any benefit in the treatment of addiction. Studies will be needed to investigate various addictions, as we have discussed in this review that the insula plays a role in a variety of drug and behavioral addictions. Also, there are currently no established parameters or treatment plans for brain stimulation for addiction, and these factors have been shown to play a major role in the outcomes of stimulation. For instance, although TMS is approved for use for major depression disorder, there is a considerable amount of variance in its effectiveness. Many studies demonstrate that it is efficacious (George et al., 1997; Klein et al., 1999; Berman et al., 2000; George et al., 2000; O’Reardon et al., 2007; George et al., 2010; Harel et al., 2014), including data from meta-analyses (Berlim et al., 2014; Mutz et al., 2018). However, there is also a large number of studies that found no beneficial effect of TMS for depression (Boutros et al., 2002; Loo et al., 2003; Carpenter et al., 2017; Yesavage et al., 2018), as well as meta-analyses (Martin et al., 2002; Couturier, 2005). Others have found that only certain variations of TMS are effective (Li et al., 2014; Blumberger et al., 2016; Brunoni et al., 2017). Given the heterogeneous outcomes in brain stimulation studies for depression, researchers will need to carefully consider the stimulation intensity, frequency, duration, and other parameters. We also described that brain stimulation has been shown to activate secondary sites (i.e., not the primary stimulation site), it is not known what other areas will be activated through this when stimulating the insula. For that reason, studies involving both brain stimulation of the insula and imaging and/or EEG are much needed and could provide a considerable amount of information. Furthermore, electric-field modeling is another tool that may be useful to estimate the insular induced electric field by TMS, thus allowing for better planning and optimization of the stimulation parameters. It is also important to consider the concept of personalized medicine. As we described under the section “A Tool for Prognosis and Relapse Prediction,” insular activation patterns and the link between the insula and striatum can be used to predict outcome. We strongly encourage future research to pursue personalized treatment plans, based on a better understanding of the individual brain activation patterns.

Individuals with mental illnesses might also benefit from insular stimulation. The role of the insula and the dACC (which compose the SN) as a common neurological substrate for mental illness was demonstrated in recent meta-analyses studies (Goodkind et al., 2015; Downar et al., 2016), and it is well known that SUDs are more prevalent in this population (Whiteford et al., 2013). Future studies should consider investigating insular stimulation as a dual treatment for symptoms of both mental illnesses and SUDs. In this context, it will also be interesting to see if stimulation of the insula improves the negative affect that is related to withdrawal.

Another factor to consider for future studies should be that of motivation to quit. A person diagnosed with a smoking-related disease is scientifically more likely to cease smoking (Twardella et al., 2006), which could be attributed to an increased motivation to quit. In addition, patients with insular lesions who live in a country with little awareness to smoking-related harm, were not more likely to quit smoking (Bienkowski et al., 2010), this finding suggests that cultural norms may influence individual motivation for quitting. Therefore, it seems that the motivation to quit and the insular damage co-influence the likelihood to cease smoking. Future studies should investigate whether insular stimulation will be more beneficial to individuals who suffer from substance abuse-related diseases. We suggest that it can be of great importance to investigate the role of motivation in patients undergoing insular stimulation, which may further increase the effectiveness of the treatment. This can be done by trying to elevate motivation to quit using patient education programs or cognitive behavioral therapy.

Furthermore, future research could explore the effect of targeting the insula using brain stimulation, as well as with pharmacotherapies. There is evidence that modafinil (Cera et al., 2014), ketamine (Carlson et al., 2013; Höflich et al., 2017; Evans et al., 2018), baclofen (Franklin et al., 2011; Franklin et al., 2012), oxytocin (Riem et al., 2011) and varenicline (Sutherland et al., 2013a) have an effect on the insula. Thus, the effect of dual targeting of the insula could be of interest. As previously mentioned, our laboratory is working on a clinical trial combining varenicline treatment with deep rTMS to the insula for smoking cessation. This will aid in expanding some of the knowledge that is lacking in this field.

An interesting question that arises from this review is to what extent are the described alterations predisposition to addiction and to what extent are they acquired during repeated drug use. Interestingly, a significant reduction in gray matter in the aIC was unique to stimulant-dependent individuals when compared with their siblings (Ersche et al., 2012). On the other hand, individuals with a family history of alcohol dependence showed alternations in insular function compared to controls (Cservenka, 2016). Although this question warrants further investigation, this discussion is not in the scope of this review.

Lastly, there are several limitations concerning the current evidence that should be noted. With regard to the clinical data, the absolute number of participants in these studies is small, and most are males. In addition, studies approached different phases of addiction (i.e., abstinent vs non-abstinent users). The methods of the studies also vary (i.e., different rsFC protocols), which could lead to variability in findings. Also, the imaging studies presented are correlational and not causative, thus making conclusions from these studies should be done with caution. It is important to note that even though there are limitations to the studies and although the majority have found other brain regions to be involved in addiction, the insula is a common area found in all of these findings, thereby further justifying it prominent role in addiction and its potential use as a target for brain stimulation in addicted individuals.

## Conclusion

In conclusion, the insula has many critical functions pertaining to addiction. Individuals with SUDs have been shown to have structural and functional changes to the insula. Also, preclinical work and human lesion studies have demonstrated that addiction is altered when there is damage to the insula. The convincing evidence leads to the idea of the insula being a promising brain region to target for addiction. Brain stimulation techniques, specifically deep TMS, are advancing and allow the targeting of the insula. This may provide promising treatment outcomes in addicted individuals. Nonetheless, there is a need for further research to determine if that is the case.

## Author Contributions

CI, DR-K, AP, and MM drafted the manuscript. DB, ZD, AZ, and BF reviewed and edited the manuscript. All authors contributed to the conception of the manuscript.

## Conflict of Interest Statement

DB has received research support from the CIHR, NIH, Brain Canada, and the Temerty Family through the CAMH Foundation and the Campbell Research Institute. He received research support and in-kind equipment support for an investigator-initiated study from Brainsway Ltd., and he is the principal site investigator for three sponsor-initiated studies for Brainsway Ltd. He received in-kind equipment support from Magventure for investigatorinitiated research. He received medication supplies for an investigator-initiated trial from Indivior. He has participated in an advisory board for Janssen. In the last 5 years, ZD has received research and equipment in-kind support for an investigator-initiated study through Brainsway Inc. and Magventure Inc. His work was supported by the Ontario Mental Health Foundation (OMHF), the Canadian Institutes of Health Research (CIHR), the National Institutes of Mental Health (NIMH), and the Temerty Family and Grant Family and through the Centre for Addiction and Mental Health (CAMH) Foundation and the Campbell Institute. AZ is an inventor of deep TMS coils which were patented by NIH and licensed to Brainsway Ltd., where these coils are further developed and applied for commercial usage. He also serves as a consultant for and has financial interest in Brainsway Ltd. BF has received in-kind support from Brainsway Ltd. that provided him with the H-coil and has been awarded the Global Research Awards for Nicotine Dependence (GRAND) from Pfizer Inc. AP is employed by Ironstone Product Development and holds stock in, as well as serves on, the Board of Directors for, Qunuba Sciences Inc. MM is employed by Ironstone Product Development.

The remaining authors declare that the research was conducted in the absence of any commercial or financial relationships that could be construed as a potential conflict of interest.

## Footnotes

1 Of note, there are currently no brain stimulation techniques approved by Health Canada or the U.S. Food and Drug Administration (FDA) for the treatment of addiction.
